# Vision 2040 in Japan: renewing UHC through community-based health care and health security

**DOI:** 10.1016/j.lanwpc.2026.101944

**Published:** 2026-07-31

**Authors:** The Lancet Regional Health – Western Pacific

Japan's health-care system is widely regarded as a successful model of universal health coverage (UHC), reflected in part by one of the highest life expectancies in the world. Japan continues to achieve high levels of UHC service coverage (85.5/100 in 2023), with particularly strong performance in infectious disease services and health-care access. However, weaker performance in non-communicable diseases and reproductive, maternal, newborn, and child health, together with persistent out-of-pocket financial hardship, highlights growing challenges in meeting the needs of an ageing population, increasing chronic disease burden, multimorbidity, and demand for integrated and long-term care. Lonely deaths among older adults have become increasingly common in Japan. The Cabinet Office recently estimated that 22,222 people were found dead at home more than 8 days after death, of whom 70% were aged 65 years or older, highlighting the growing social consequences of population ageing and additional workload and emotional strain on local governments.

Health challenges in Japan do not occur in isolation. They unfold with a rapidly changing regional and global landscape characterised by demographic transitions, climate change, and geopolitical uncertainty. A systematic review and scenario-based analysis from Japan found that current financial incentives have only a 12% likelihood of reversing fertility decline by 2030 and a 29% likelihood by 2035, highlighting the need for broader health-system, social, economic, and structural reforms. Yamasaki and colleagues propose *Vision 2040* in Japan: a transition from a hospital-centred system to a community-based model that supports people to live well in their communities. This vision reflects a life-course approach that focuses on prevention, integrated care, and wellbeing across the lifespan, which might better support older adults to live independently and remain connected to their communities. Such a vision rests on evidence-based governance, with clearly defined public–private responsibilities, safeguards for scientific independence, and mandated transparency and accountability in policy making. Additionally, a unified health and social data infrastructure to inform resource allocation that considers social determinants of health and societal value could support early identification of populations vulnerable to climate-related health risks, increase investment in prevention and community care, and facilitate regional partnerships to strengthen the health workforce.

In their review of Japan's global health engagement in the Western Pacific region, Sakamoto and colleagues propose that Japan should view health not only as a domestic priority but also as an integral part of its human security diplomacy. In their opinion, health underpins economic security and strengthens regional cooperation and resilience to global challenges such as climate change and pandemics. Achieving this would entail protecting long-term funding for health programmes from budget cuts, similar to funding models for national defence or disaster preparedness, stronger alignment of global health investments and national priorities (eg, population ageing and pandemic preparedness), and governance structures and digital systems that better connect health and social sectors to facilitate information sharing and coordinated efforts (eg, Association of Southeast Asian Nations-developed mHealth platforms and artificial intelligence-assisted diagnostics). Finally, a shift from measuring success by how much money is spent or how many programmes are funded to real-world impact assessments (eg, disability-adjusted life years averted) would position global health expenditure as a strategic investment in mutual security and risk reduction.

Japan is a pioneer of UHC and is well positioned to shape the next generation of health-system and global health reforms. Its experience serves as an important reminder that future health policy must adopt an integrated, life-course approach. Policy silos are ultimately unsustainable as demographic, environmental, and geopolitical challenges are increasingly intertwined. These challenges cannot be fully understood or effectively addressed in isolation if UHC is to advance now and in the future.

